# The impact of psychostimulants on central and peripheral neuro-immune regulation: a scoping review of cytokine profiles and their implications for addiction

**DOI:** 10.3389/fncel.2023.1109611

**Published:** 2023-05-26

**Authors:** Joana Bravo, Catarina Magalhães, Elva B. Andrade, Ana Magalhães, Teresa Summavielle

**Affiliations:** ^1^Addiction Biology, i3S-Instituto de Investigação e Inovação em Saúde, Universidade do Porto, Porto, Portugal; ^2^IBMC—Instituto de Biologia Molecular e Celular, Universidade do Porto, Porto, Portugal; ^3^ICBAS—Instituto de Ciências Biomédicas de Abel Salazar, Universidade do Porto, Porto, Portugal; ^4^Escola Superior de Saúde, Polytechnic of Porto, Porto, Portugal; ^5^Centro Hospitalar Vila Nova de Gaia/Espinho, Vila Nova de Gaia, Portugal; ^6^Immunobiology, i3S-Instituto de Investigação e Inovação em Saúde, Universidade do Porto, Porto, Portugal; ^7^Instituto Universitário de Ciências da Saúde, Cooperativa de Ensino Superior Politécnico e Universitário (CESPU), Gandra, Portugal

**Keywords:** methamphetamine, cocaine, methylphenidate, amphetamine, acute-use, chronic-use, withdrawal, reinstatement

## Abstract

It is now well-accepted that psychostimulants act on glial cells causing neuroinflammation and adding to the neurotoxic effects of such substances. Neuroinflammation can be described as an inflammatory response, within the CNS, mediated through several cytokines, reactive oxygen species, chemokines and other inflammatory markers. These inflammatory players, in particular cytokines, play important roles. Several studies have demonstrated that psychostimulants impact on cytokine production and release, both centrally and at the peripheral level. Nevertheless, the available data is often contradictory. Because understanding how cytokines are modulated by psychoactive substances seems crucial to perspective successful therapeutic interventions, here, we conducted a scoping review of the available literature. We have focused on how different psychostimulants impact on the cytokine profile. Publications were grouped according to the substance addressed (methamphetamine, cocaine, methylphenidate, MDMA or other amphetamines), the type of exposure and period of evaluation (acute, short- or long-term exposure, withdrawal, and reinstatement). Studies were further divided in those addressing central cytokines, circulating (peripheral) levels, or both. Our analysis showed that the classical pro-inflammatory cytokines TNF-α, IL-6, and IL-1β were those more investigated. The majority of studies have reported increased levels of these cytokines in the central nervous system after acute or repeated drug. However, studies investigating cytokine levels during withdrawal or reinstatement have shown higher variability in their findings. Although we have identified fewer studies addressing circulating cytokines in humans, the available data suggest that the results may be more robust in animal models than in patients with problematic drug use. As a major conclusion, an extensive use of arrays for relevant cytokines should be considered to better determine which cytokines, upon the classical ones, may be involved in the progression from episodic use to the development of addiction. A concerted effort is still necessary to address the link between peripheral and central immune players, including from a longitudinal perspective. Until there, the identification of new biomarkers and therapeutic targets to envision personalized immune-based therapeutics will continue to be unlikely.

## 1. Introduction

Addiction is a chronic complex disease in which, after a period of episodic drug use, a subset of individuals develops problematic drug use and use related disorders, which will likely be followed by periods of abstinence and eventual relapse (Koob and Volkow, 2010; Nestler and Luscher, 2019). The latest World Drug Report estimates 35.6 M of problematic drug-users, for whom there are limited, and often unspecific, treatment options (UNODC, 2021).

Psychoactive substances are classically recognized by their neurotoxic effects on monoaminergic and glutamatergic systems (Nestler and Luscher, 2019). However, it is now evident that these substances also promote neuroinflammation and that seems to contribute to the establishment of problematic drug use and addiction (Miguel-Hidalgo, 2009; Kohno et al., 2019). Neuroinflammation can be described as an inflammatory response within the central nervous system (CNS), which is mediated through several cytokines, reactive oxygen species, chemokines, and other inflammatory markers (DiSabato et al., 2016). These mediators, in particular cytokines, are produced by resident CNS cells, such as microglia, astrocytes and oligodendrocytes, but also by endothelial and peripherally derived immune cells (DiSabato et al., 2016). Cytokines are regulatory peptides that orchestrate signal-dependent immune responses. Upon their essential role in the regulation of immune and inflammatory responses, cytokines are critical to maintain homeostasis, immune cell development and differentiation (Deverman and Patterson, 2009; Hofer and Campbell, 2016; Becher et al., 2017). Furthermore, cytokines play an important role in synaptic plasticity, impacting different behavioral responses, such as sickness behavior, social behavior, learning and anxiety-like behavior (reviewed in Salvador et al., 2021). The production of cytokines in the CNS is under tight control and, usually, under physiological conditions, very low levels are detected (Hofer and Campbell, 2016). However, the disruption of homeostasis may significantly impact cytokine production and release (Hofer and Campbell, 2016; Becher et al., 2017). Although, the initial cytokine release response, may be beneficial to counteract homeostatic imbalance, the production of cytokines over long periods of time may perpetuate an adverse environment and contribute to disease and neurodegenerative processes (Becher et al., 2017).

Cytokines' action on their target cells can be autocrine (signaling to self), paracrine (signaling to neighboring cells) or endocrine (signaling through the circulation) (Altan-Bonnet and Mukherjee, 2019). Cytokines play also an important role in the communication between the peripheral and central nervous compartments (Croese et al., 2021). This direct communication is more likely to occur under dysfunctional/disease states, in which the blood brain barrier (BBB) is disrupted (Croese et al., 2021; Salvador et al., 2021). However, there is growing evidence that even in the absence of higher BBB permeability, cytokines derived from immune cells residing in the immunological niches in CNS, reach the brain parenchyma and influence resident cells and behavior (Alves de Lima et al., 2020; Croese et al., 2021; Salvador et al., 2021).

Several studies demonstrated that psychostimulants impact on cytokine production and release, both in the CNS and at the peripheral level (Coelho-Santos et al., 2015; Mata et al., 2015; Pianca et al., 2017; Canedo et al., 2021). Still, little is yet known on the peripheral and central immune crosstalk under exposure to psychostimulants. Of note, one of the hallmarks of psychostimulants' neurotoxicity is BBB dysfunction (Sajja et al., 2016), which most likely favors the entry of cytokines from the periphery into the brain parenchyma (Croese et al., 2021; Salvador et al., 2021).

In humans, a recent study addressed the associations between psychological distress linked to alcohol/drug use and circulating cytokines (Martinez et al., 2018). Distress and anxiety are both recognized as important factors for drug relapse (Willinger et al., 2002; Engel et al., 2016), but the relation between cytokines and relapse was only investigated in problematic alcohol users, where stress-related suppression of tumor necrosis factor alpha (TNF-α) predicted drinking severity (Fox et al., 2020).

In this scenario, mapping the cytokine profile at each stage of exposure (acute or chronic), withdrawal and relapse, and understand the interplay between the peripheral and central components of the immune system seems crucial to perspective new biomarkers, personalized immune-based therapeutics and more efficient treatments.

This scoping review aims at clarifying the current state of knowledge regarding the impact of psychostimulants on cytokine levels, both in the CNS and at the peripheral level, throughout the different phases of drug use. Simultaneously, it addresses a possible parallel between crucial immune players at central and peripheral compartments under exposure to psychostimulants.

## 2. Methods

The literature search was conducted following the PRISMA guidelines without a previous registered protocol (Tricco et al., 2018). We have included studies meeting the following eligibility criteria: (a) published within the last 10 years (from 2012 to June 2022); (b) redacted in English; (c) with an abstract available; (d) addressing cytokine levels either in humans or *in vivo* animal models; (e) published in journals from quartile (Q)1 or Q2, according to the Scimago Journal & Country Rank (SJR).

**Figure 1:**
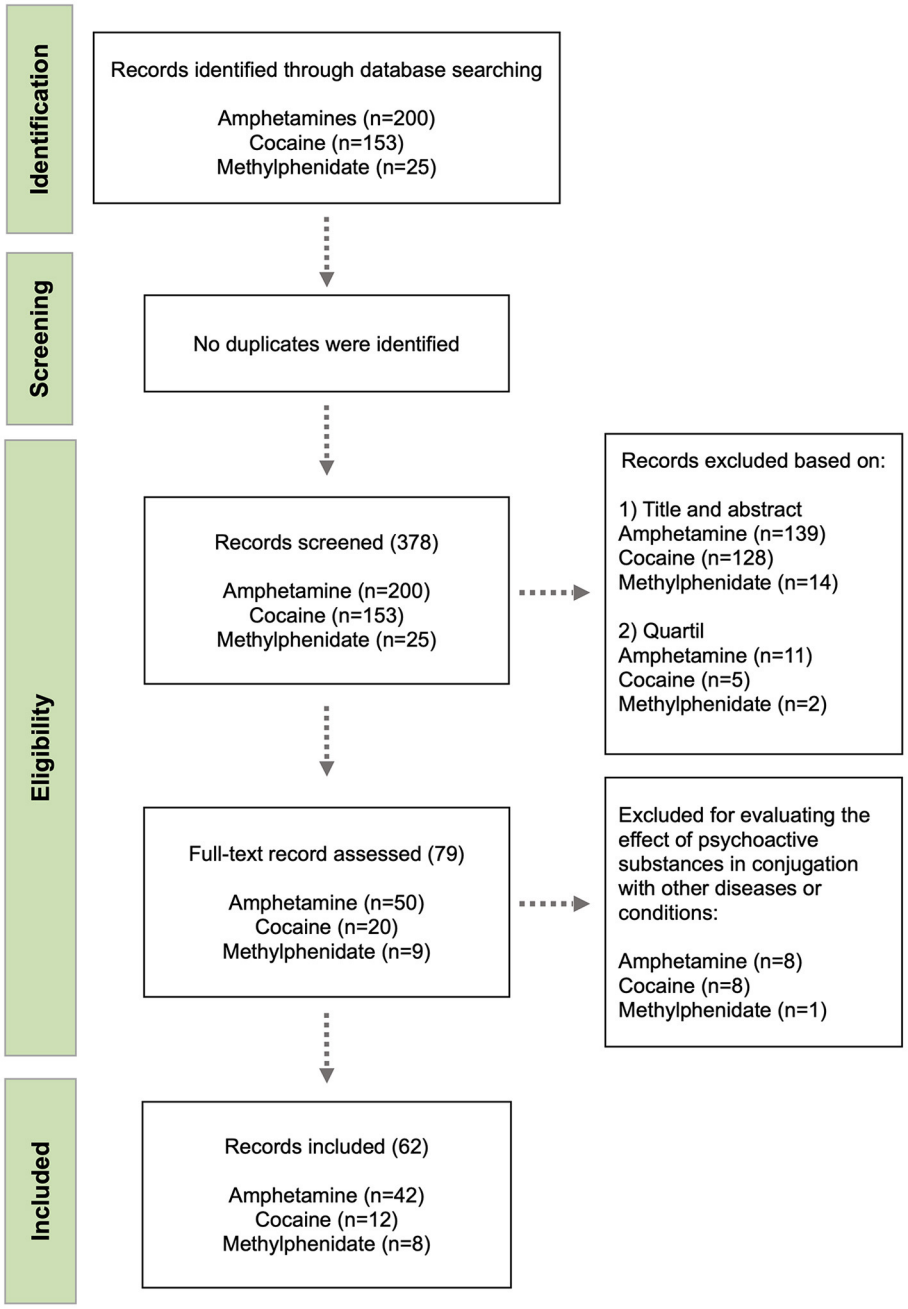
Flow diagram of the study selection process.

Our search was conducted using the Pubmed^®^, which is maintained by the National Center for Biotechnology Information (NCBI), at the U.S. National Library of Medicine (NLM), and is located at the National Institutes of Health (NIH). This database allows searching MEDLINE, PubMed Central (PCM) and Bookshelf databases and covers more than 35 million citations and abstracts of biomedical literature, providing liking access to the full-text. The following search equation was used: “(substance) AND (cytokine OR interleukin OR interferon OR ‘tumor necrosis factor’) NOT (culture),” where “substance” was replaced by amphetamine, cocaine or methylphenidate on three independent searches. A total of 200 publications for amphetamines, 153 for cocaine and 25 for methylphenidate were identified. Publications obtained in these searches were then checked to confirm that they met the eligibility criteria. Next, charting was conducted in parallel by two of the authors and differences in the initial selection were further discussed and compatibilized. At this stage, full abstracts and texts were read and the following exclusion criteria were adopted: (a) the study evaluated the conjugated effects of different psychoactive substances; (b) the study investigated drug effects after prenatal exposure; (c) the study was not empirical; (d) the study evaluated the effect of psychoactive substances in conjugation with other diseases or conditions. After this analysis a total of 50 articles for amphetamines, 20 for cocaine and nine for methylphenidate were elected for full-text reading and further confirmation. A final list of 42 publications for amphetamines, 12 for cocaine and eight for methylphenidate was used in this review. The complete selection process was represented in a flow diagram (Figure 1).

After full-text analysis, final items were further divided in three groups: the first group included articles that evaluated cytokines in the CNS; the second group included articles which evaluated peripheral cytokines; and the third group included articles that evaluated both central and peripheral cytokines. In each of these groups, data regarding: (i) subjects studied; (ii) drug dosing regimen, (iii) time-point of evaluation; (iv) type of tissues/samples evaluated; (v) methodology used for evaluation; and (vi) results obtained for the different cytokines were collected and organized by psychoactive substance in different Tables (Tables 1–5 and Supplementary Tables 1–3).

**Table 1:** Studies addressing the impact of methamphetamine in central cytokines.

	**References**	**Subjects**	**Dose regimen**	**Evaluation**	**Brain region**	**Results**	**Methodology**
Acute	Canedo et al., 2021	Adult male C57BL/6J mice	4 × 5 mg/Kg, 2 h apart, i.p.	24h after the last injection	Hippocampus Striatum	Hippocampus =TNF-α and IL-6 ↑ IL1-β Striatum =IL-6 and IL-1β ↑ TNF-α	RT-PCR
	Coelho-Santos et al., 2015	Adult male C57BL/6J mice	4 × 10 mg/kg, 2 h apart, i.p.	2 h after the last injection	Striatum	↑ TNF-α	Immunohistochemistry
	DiCaro et al., 2019	Adult male C57BL/6J mice	5 mg/Kg, i.v.	4 h after administration	Brain	= IL1-α, IL-1β, IL-6, and TNF-α	RT-PCR
	Frank et al., 2016	Adult male Sprague Dawley rats	10 mg/Kg, i.p.	2, 4, and 6 h after administration	Nacc VTA PFC	Nacc and PFC ↑ IL-1β and TNF-α at 2 h and IL-6 at 4 h VTA ↑ IL-1β, TNF-α, and IL-6 at 2 h	RT-PCR
	Ghanbari et al., 2019	Male Wistar rats	4 × 10 mg/Kg, 2 h apart, i.p.	7 days after administration	Hippocampus	↑ TNF-α	ELISA
	Gou et al., 2020	Male C57BL/6J mice	4 × 10 mg/Kg, 3 h apart, i.p.	(not reported)	mPFC CPu Hippocampus	mPFC, Cpu, and hippocampus ↑ IL-6 and TNF-α	Flow cytometric bead array
	Hadizadeh-Bazaz et al., 2021	Male Wistar rats	4 × 10 mg/Kg, 2 h apart, i.p.	12 days after administration	Hippocampus	↑ TNF-α	ELISA
	Kelly et al., 2012	Male C57BL/6J mice	20 mg/Kg, s.c.	12 h after Meth injection	Striatum, hippocampus and PFC	Striatum ↑ TNF-α, IL-6, and IL-1β Hippocampus and PFC: = TNF-α, IL-6, and IL-1β	RT-PCR
	Nader et al., 2014	Male C57BL/6J mice	30 mg/Kg, i.p.	24 h after administration	Striatum	↑ TNF-α	ELISA
	Robson et al., 2013	Male Swiss Webster mice	4 × 5 mg/Kg, 2 h apart, i.p.	1.5; 3; 6; 12 and 24 h after administration	Striatum	↑ IL-6 (all timepoints)	RT-PCR
	Seminerio et al., 2012	Male Swiss Webster mice	25; 35 or 45 mg/Kg, i.p.	45 min after administration	Hypothalamus, striatum and PFC	Hypothalamus and striatum ↑ IL-1β (35 and 45 mg/Kg) PFC ↑ IL-1β (45 mg/Kg)	RT-PCR
	Urrutia et al., 2014	C57BL/6J mice	3 × 4 mg/Kg, 3 h apart, i.p.	24 h after administration	Striatum	↑ IL-15	Immunohistochemistry
	Wang B. et al., 2019	Male C57BL/6J mice	4 × 5 mg/Kg, 2 h apart, i.p.	24 h after the first administration	Hippocampus, PFC, CPU and Nacc	= IL-6 and TNF-α	ELISA
	Wang J. et al., 2019	Male Sprague-Dawley rats	A single injection of 1 mg/Kg, i.p.	30 min and 2 h after injection	VTA, Nacc and PFC	VTA ↑ TNF-α (at 30 min) and IL-6 (both time points) = TNF-α (at 2 h) Nacc and PFC = TNF-α and IL-6	RT-PCR
Short- and long-term administration	Liskiewicz et al., 2019	Male C57BL/6NCrL mice	Escalating doses (0.2–2.4 mg/Kg, using an increase step-wise of 0.2 in each injection, 3 × per day for 4 days). Then binge administration (3 × 4 mg/Kg, 3 h apart, i.p.)	24 h after last Meth dose	Hippocampus	↑ IL-1β = IL-6	ELISA
	Goncalves et al., 2017	Adult male Wistar rats	Self-administration (Meth 0.1mg/Kg per infusion. From day 1 to day 3, the sessions lasted 2 h or 25 infusions. From day 4 to day 10, the sessions lasted 6 h or 50 infusions)	24 h or 7 days after the last operant session	Hippocampus	Hippocampus ↑TNF-α (at 24 h and 7 days) = IL-1β Striatum ↑ TNF-α (at 24 h and 7 days) ↑ IL-1β (at 24 h) ↓ IL-1β (at 7 days)	Western blot
					Striatum		
Withdrawal	Beirami et al., 2017	Adult male Wistar rats	Escalating doses (1–10 mg/kg; twice a day, at 5-h intervals, for 10 consecutive days, i.p.)	2 weeks after last administration	Hippocampus	↑ TNF-α and IL-6	Western blot
	Jiang et al., 2014	Adult male Wistar rats	20 mg/Kg for 5 days, i.p.	4, 6, 10 and 14 days after the initial injection	Substantia nigra	Day 4: = IL1-β and TNF-α Day 6, 10, and 14: ↑ IL-1β, and TNF-α	ELISA
	Loftis et al., 2013	Male C57BL/6J mice	4 mg/Kg once daily for 15 consecutive days, s.c.	6 days after last Meth dose	Hypothalamus	↑ IL-2 = IFN-γ, TNF-α, IL-6, IL-1β, and IL-10	Multiplex immunoassay
	Namyen et al., 2020	Wistar rats	Increasing doses (days 1 and 2–2.5, days 3 and 4–5 and days 5 to 11–10 mg/Kg)	On day 15	Hippocampus and PFC	↑ IL-1β; IL-6, and TNF-α	Western blot and RT-PCR
	Stolyarova et al., 2015	Long Evans rats	Increasing doses (0.3–6 mg/Kg with 0.3 increments/day, in 5 days per week for 4 weeks, s.c.)	17 days after the last administration	Frontal cortex; amygdala and striatum	Frontal cortex ↓ IL-1β, IL-6, IL-10, and TNF- Amygdala and striatum = IL-1β, IL-6, IL-10, and TNF-α	ELISA
Reinstatement	Karimi-Haghighi et al., 2020	Male Wistar rats	Conditioned place preference paradigm (5 days of consecutive conditioning with 1 mg/Kg, s.c.. Followed by 10 days of extinction and then a reinstatment day with Meth 0.25 mg/Kg or 0.5 mg/kg, s.c.)	Immediately after the reinstatement behavior test	PFC and hippocampus	PFC Reinstatment with 0.25 = TNF-α, IL-6, and IL-10 ↑ IL-1β Reinstatment with 0.5 ↑ TNF-α, IL-1β, and IL-10 = IL-6 Hippocampus Reinstatment with 0.25 = TNF-α, IL-1β, IL-6, and IL-10 Reinstatment with 0.5 ↑ TNF-α, and IL-10 = IL-1β and IL-6	RT-PCR

CPu, caudate putamen; ELISA, enzyme-linked immunosorbent assay; IFN, interferon; IL, interleukin; i.p., intraperitoneal; i.v., intravenous; Meth, methamphetamine; mPFC, medial Prefrontal cortex; Nacc, nucleus accumbens; PFC, prefrontal cortex; RT-PCR, real time polymerase chain reaction; s.c., subcutaneous; TNF-α, tumor necrosis factor α; VTA, ventral tegmental area.

Results are expressed relative to control (↑, increase; ↓, decrease; =, unaltered).

**Table 2:** Studies addressing the impact of methamphetamine in peripheral cytokines.

	**References**	**Subjects**	**Dose regimen**	**Evaluation**	**Sample**	**Results**	**Methodology**
Acute	Kobeissy et al., 2022	Male Sprague Dawley rats	4 × 10 mg/Kg, 1 h apart, i.p.	24 h after injection	Serum	Cytokine antibody kit: ↑ IL-1β, IL-6, and IL-10 ELISA: ↑ IL-6 and IL-10	Cytokine antibody kit ELISA
Short- and long-term	Jiang et al., 2016	Male and female Meth users (15) and healthy controls (15) from the same Chinese cities	Chronic Meth use history longer than 1 year (use within 3 months and no other drugs use within 3 months)	Subjects with a positive result for recent Meth use (72 h) and an history of at least an year	PBMCs and plasma	PBMCs and plasma = IL-6 PBMCs ↑ IFN-α	RT-PCR ELISA
	Mata et al., 2015	Sprague-Dawley rats	Self-administration (Meth 0.1 mg/Kg per infusion. FR1 From day 1 to day 7. FR5 from day 8 to 14)	One day after the last operant session	Serum	= TNF-α, IL-6, and IFN-γ	ELISA
	Shen et al., 2020	Meth-dependent patients (380 male and 27 female) from rehabilitation centers and hospital in Kunming (China)	Participants met criteria for Meth dependence. Mean duration of dependency: 114.37 ± 106.752 months	Before detoxification treatment	Serum	↑ TNF-α	ELISA
	Wang et al., 2022	Male C57BL/6J mice	8 Meth injections at a rate of 1.5 to 10 mg/Kg, i.p.	24 h after the last injection	Serum	↑ TNF-α, IL-1β, and IL-18	ELISA
Withdrawal	Kuo et al., 2018	Female patients with amphetamine dependence from drug rehabilitation clinic in a detention center in Northern Taiwan (72); healthy controls (51)	Participants met DSM-IV criteria for Meth dependence	Samples were collected < 3 days after last drug use and after 4-week abstinence	Plasma	At admission: ↑ IL-1β, IL-2, IL-4, IL-6, IL-10 = IFN-γ, TNF-α, and IL-8 ↓ IL-5 At abstinence: ↑ IL-1β, IL-2, IL-4, and IL-6 = IFN-γ, TNF-α, and IL-5 ↓ IL-8 and IL-10	Multiplex immunoassay
	Kohno et al., 2018	Male and female Meth users (30) and healthy controls (20) recruited from the community and treatment centers.	Participants met DSM-IV criteria for Meth dependence and had been abstinent for > 1 month and < 6 months	Abstinent from Meth for >1 month and < 6 months	Plasma	= IL-1β and IL-10 ↑ IL-6	Multiplex immunoassay
	Li et al., 2021	Male Sprague-Dawley rats	Conditioned place preference paradigm (2 mg/Kg daily for 10 days, i.m.)	18 days after Meth administration	Serum	↑ IL-1α and IL-2	ELISA
	Luo et al., 2022	In patients (78) from voluntary drug rehabilitation hospital Guangzhou Baiyun (male and female); healthy controls (64)	Participants met DSM-IV criteria for Meth dependence	39.06 ± 7.48 days from the last use	Serum	↑ TNF-α, IL-6, and IL-18	ELISA
	Re et al., 2022	Meth users (67) recruited at the Yunnan drug rehabilitation center; 38 healthy matched controls	Participants met DSM-V criteria for Meth dependence.	7–15 days (M-0.5); 3 months (M-3) and 12 months (M-12) after withdrawal	Plasma	↑ IL-6, IL-12p70, IFN-γ, TNF-α, and IL-7 (showed a downward trend over the withdrawal time) ↓ IL-9 and IL-1β (M−0.5 and M−3) ↑ IL-2 and IL-5 (M-0.5 and M-3) ↑ IL-10, IL-4 and IL-5 (M-12)	Bio-Plex assay kit
Reinstatement	Li et al., 2020	Male (9) and female (2) suffering from Meth use disorder	Participants met DSM-IV criteria for Meth dependence and tested positive for Meth at least once prior to admission (urine tested). Patients received a 30 mg Meth intravenous (i.v.) challenge delivered over 2 min	Blood was collected at 0, 60 and 360 min post-Meth infusion	Plasma	= TNF-α ↑ IL-6 after 360 min (but not 60 min)	Multiplex immunoassay

DSM, diagnostic and statistical manual of mental disorders; ELISA, enzyme-linked immunosorbent assay; FR, fixed ratio; IFN, interferon; IL, interleukin; i.m, intramuscular; i.p., intraperitoneal; i.v., intravenous; Meth, methamphetamine; PBMCs, peripheral blood mononuclear cells; RT-PCR, real time polymerase chain reaction; s.c., subcutaneous; TNF-α, tumor necrosis factor α.

Results are expressed relative to control (↑, increase; ↓, decrease; =, unaltered).

**Table 3:** Studies addressing the impact of cocaine in central cytokines.

	**References**	**Subjects**	**Dose regimen**	**Evaluation**	**Brain region**	**Results**	**Methodology**
Acute	Montesinos et al., 2020	Male OF1 mice	Acute: single injection of 25mg/Kg Repeated: 25 mg/Kg daily, for 7 days, i.p.	0, 30, 60, 120, and 240 min after cocaine injection	Hippocampus	↑ IL-1β (in repeated model, at 60 min) = IL-1β (in other time-points and in the acute model)	ELISA
	^*^Lewitus et al., 2016	C57BL/6 mice	Single injection of 15 mg/Kg or 5 daily injections of 15 mg/Kg	24 h later after single injection or after the last injection. And 10 days after 5 daily injections	Ventral striatum	↑ TNF-α (24 h later of 5 daily injections) = TNF-α (at single injection and after 10 days withdrawal)	Immunohistochemistry RT-PCR
Short- and long-term	Chivero et al., 2021	C57BL/6 mice Cocaine users (postmortem)	20 mg/Kg daily, for 7 consecutive days Chronic cocaine dependence	1 h after the last administration	Striatum and cortex Frontal cortex	↑ IL-1β	Western blot
	Mai et al., 2018	C57BL/6 mice	45 mg/Kg/day, for 5 days, i.p.	1 h, 6 h, 12 h, 1 day, 3 days, 7 days and 14 days after the last dose	Hippocampus	↑ IL-6 (all time points) ↓ IFN-γ (at 12 h, 1 and 3 days) ↑ TNF-α (at 1, 6, and 12 h)	RT-PCR Western blot
Withdrawal	Zhu et al., 2018	Male Sprague-Dawley adolescent rats	Single daily injection of 15 mg/Kg, during 15 days, i.p.	After 35 days	mPFC	= IL-6, IL-1β, and TNF-α	Western blot
Reinstatement	Brown et al., 2018	Male Sprague-Dawley rats	Self-administration (0.5 mg/Kg/infusion, during 2 h daily sessions over 15 days). After self-administration rats received seven 2 h extinction sessions. After drug-extinction, rats received a drug priming stimulus of 15 mg/Kg, i.p.	2 h after cocaine challenge	VTA	↑ IL-1β = TNF-α	RT-PCR

ELISA, enzyme-linked immunosorbent assay; IFN, interferon; IL, interleukin; i.p., intraperitoneal; mPFC, medial prefrontal cortex; RT-PCR, real time polymerase chain reaction; TNF-α, tumor necrosis factor α; VTA, ventral tegmental area.

Results are expressed relative to control (↑, increase; ↓, decrease; =, unaltered).

* Also report withdrawal data.

**Table 4:** Studies addressing the impact of cocaine in peripheral cytokines.

	**References**	**Subjects**	**Dose regimen**	**Evaluation**	**Sample**	**Results**	**Methodology**
Short- and long-term	Ribeiro et al., 2021	Male (12) chronic users (or not) from a prison the State of Goias, Brazil	Chronic users. Urine positive for cocaine	In blood samples positive for cocaine	Serum	= TNF-α, IL-6, and IL-10	ELISA
	Pianca et al., 2017	Adolescents aged 12–18 years	Participants met DSM-IV criteria for cocaine (crack) dependence	On admission and 21 days later	Serum	↑ IL-6 and IL-10 (on admission) = IL-6 and IL-10 (after 21 days)	Flow cytometric bead array
Withdrawal	Zaparte et al., 2019	Female (50) from an in patient unit from Southern Brazil suffering from CUD	Participants met DSM-V criteria for cocaine (crack) dependence	4 days after the initiation of the detoxification treatment	PBMCs	↑ TNF-α, IFN-γ, IL-4, and IL-10 (low and high withdrawal) ↑ IL-2 and IL-17 (low withdrawal) ↑ IL-6 (high withdrawal)	Flow cytometric bead array
	Levandowski et al., 2016a	Women (36) recruited from therapeutic communities in Southern Brazil	Participants met DSM-IV criteria for cocaine (crack) dependence. No acute abstinence (>20 days)	At admission	Serum	↑ IL-6	Flow cytometric bead array
	Levandowski et al., 2016b	Women (108) recruited from therapeutic communities in Southern Brazil	Participants met DSM-IV criteria for cocaine (crack) dependence	On day 4, 11 and 18 of the 3-week detoxification period	Serum	↓ TNF-α and IL-6 (day 4 and 11) = TNF-α and IL-6 (day 18) ↓ IFN-γ, IL-2, and IL-17A ↑ IL-4 and IL-10	Flow cytometric bead array
Reinstatement	Gupta et al., 2018	Male adults (11) with current cocaine use	Cocaine users within the past 4 week After 24 h of admission, subjects received a cocaine bolus at 0.18 mg/Kg and if tolerated then 90 min later another cocaine bolus at 0.36 mg/Kg, i.v.	Before cocaine injection and 6 h, 24 h and 6 days after cocaine challenge	Serum	= IL-6	ELISA

CUD, cocaine use disorder; DSM, diagnostic and statistical manual of mental disorders; ELISA, enzyme-linked immunosorbent assay; FACS, fluorescence activated cell sorting; IFN, interferon; IL, interleukin; i.v., intravenous; PBMCs, peripheral blood mononuclear cells; TNF-α, tumor necrosis factor α.

Results are expressed relative to control (↑, increase; ↓, decrease; =, unaltered).

**Table 5:** Studies addressing the impact of methylphenidate in central cytokines.

	**References**	**Subjects**	**Dose regimen**	**Evaluation**	**Brain region**	**Results**	**Methodology**
Short- and long-term	Ebrahimzadeh et al., 2019	Male Wistar rats	10 mg/Kg for 21 days, i.p.	On day 22	Hippocampus	↑ IL-1β and TNF-α	ELISA
	Motaghinejad and Motevalian, 2016	Male rats	10 mg/Kg for 21 days, i.p.	On day 22	Hippocampus	↑ IL-1β and TNF-α	ELISA
	Motaghinejad and Motevalian, 2022	Male Wistar rats	10 mg/Kg for 21 days, i.p.	On day 22	Hippocampus	↑ IL-1β and TNF-α	ELISA
	Motaghinejad et al., 2016a	Male Wistar rats	2, 5,10 or 20 mg/Kg for 21 days, i.p.	On day 22	Hippocampus	↑ IL-1β and TNF-α (using 10 or 20 mg/Kg for 21 days)	ELISA
	Motaghinejad et al., 2016b	Male Wistar rats	10 mg/Kg for 21 days, i.p.	On day 22	Hippocampus	↑ IL-1β and TNF-α	ELISA
	Motaghinejad et al., 2017	Male Wistar rats	2, 5,10 or 20 mg/Kg, i.p.	On day 22	Hippocampus	↑ TNF-α ↑ IL-1β (with 10 and 20 mg/Kg dose regimen)	ELISA
	Motaghinejad et al., 2016c	Male Wistar rats	10 mg/Kg for 28 days, i.p.	24 h after the last administration	Hippocampus	↑ IL-1β and TNF-α	ELISA
	Schmitz et al., 2017	Juvenile male Wistar rats	2 mg/Kg for 30 days, i.p.	24h after the last administration	Hippocampus	↑ IL-6 and TNF-α	ELISA

ELISA, enzyme-linked immunosorbent assay; IL, interleukin; i.p., intraperitoneal; TNF-α, tumor necrosis factor α.

Results are expressed relative to control (↑, increase).

## 3. Results

### 3.1. Overview

The articles included in this study for full review cover a range of publications addressing the evaluation of several cytokines both at central and peripheral levels, in animal models and in studies conducted in patients with problematic drug use. These studies also cover different stages of the addictive process, extending from acute drug exposure, to short- or long-term administration/use, withdrawal and drug reinstatement. For a comprehensive understanding of the results, they were grouped according to the type of substances administered/used and according to the type of administration and period evaluated.

**Figure 2:**
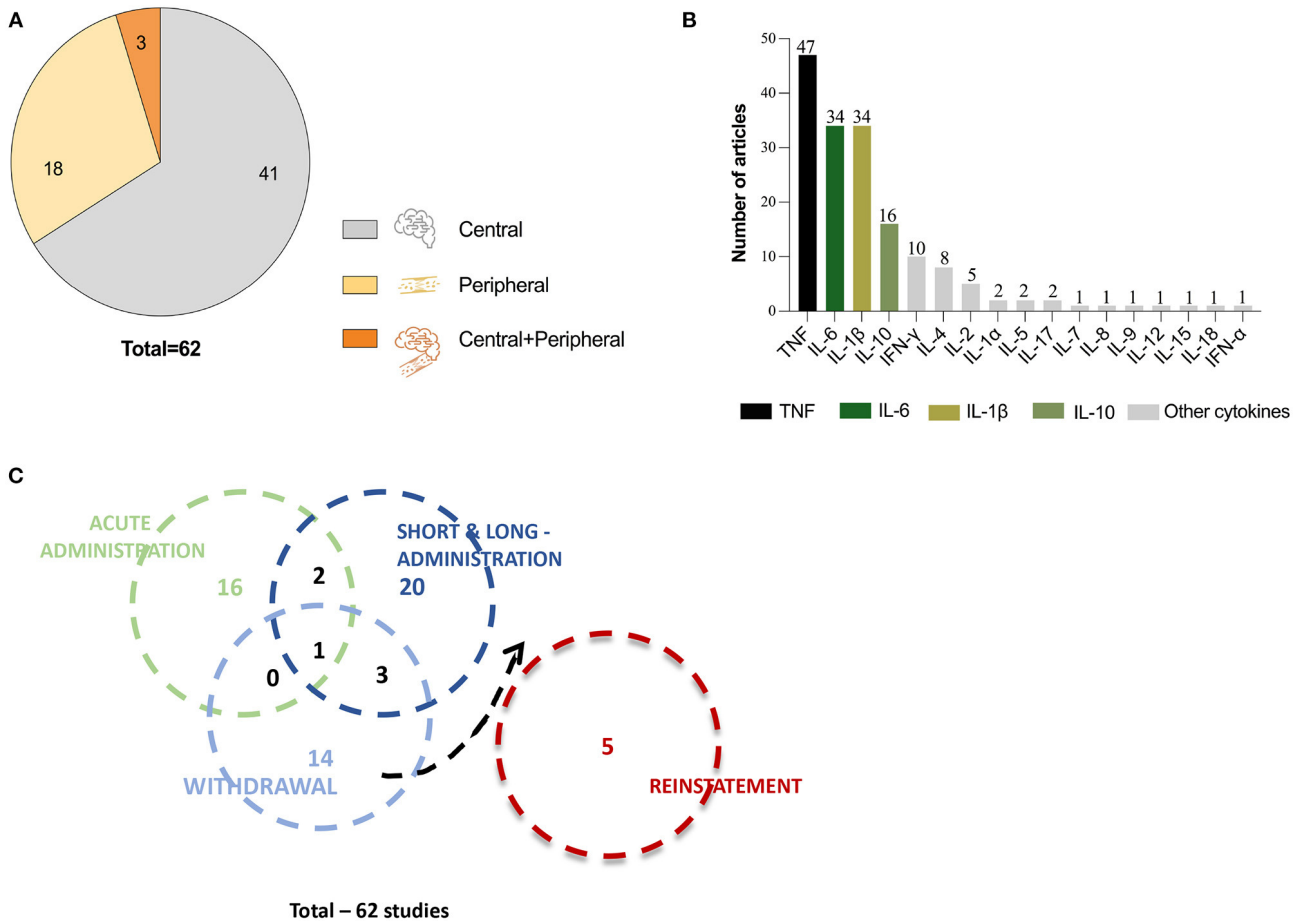
Overview of the 62 studies included for revision. **(A)** Pie chart representing the total number of articles included for revision and divided in three compartments that correspond to the number of articles that evaluated cytokines at central level (gray), at peripheral level (light orange) and at central and peripheral level simultaneously (dark orange). **(B)** Graphic representation of all cytokines evaluated in the reviewed articles and the number of articles that evaluated each cytokine. **(C)** Venn diagram showing distribution of the number of studies performed at acute, short- and long-term administration, withdrawal and reinstatement, represented by circles. The circle overlapping shows the number of studies that evaluated cytokine levels in multiple timepoints or stages.

After full-text analysis, a pool of 62 publications were considered eligible. Within these, we found 41 publication that evaluated cytokines in the CNS and 18 publications that evaluated cytokines at the peripheral level (Figure 2A). Only three studies evaluated cytokines both at central and peripheral level (Figure 2A). We also analyzed the distribution of cytokines in all the elected studies and found that 17 different cytokines had been evaluated in at least one study (Figure 2B), while most studies addressed several cytokines simultaneously. As expected, the most studied cytokines were the classical pro-inflammatory cytokines TNF-α, IL-6, and IL-1β. The TNF-α profile was evaluated in 47 articles, while IL-6 and IL-1β were both evaluated in 34 articles. As for IL-10, IFN-γ, IL-4, and IL-2, they were evaluated in 16, 10, eight, and five articles, respectively. The cytokines Il-1α, IL-5, and IL-17 were investigated in only two studies. The remaining cytokines—IL-7, IL-8, IL-9, IL-12, IL-15, IL-18, and IFN-α—were addressed in just one publication each (Figure 2B).

To analyze the results reported in the different studies, we have categorized them by type of exposure and/or evaluation timepoint, which allowed four different categories addressing: (i) acute exposure (19 publications); (ii) chronic (mid- or long-term) exposure (27 publications); (iii) withdrawal/abstinence (nine publications); and (iv) reinstatement (five publications; Figure 2C). This categorization is reflected in Tables 1–5 and Supplementary Tables 1–3 and described in detail in the following subsections.

For reading simplification, significantly altered cytokines are referred to as increased/decreased (or equivalent adjectives), however, this implies always that a comparison to an adequate control group was verified.

### 3.2. Cytokine profile in acute administration of psychostimulants

Drug use often refers to episodic or recreational administration of psychoactive substances (Kuhar, 2012). The effects of acute exposure to psychostimulants can either refer to the adverse effects that result from a single dose or from multiple dosing in a short period of time (i.e., binge drug exposure). Among the 19 studies that evaluated the acute effects of psychostimulants in the cytokine profile, nine used a protocol of a single dose administration (Kelly et al., 2012; Seminerio et al., 2012; Nader et al., 2014; Frank et al., 2016; Gubert et al., 2016; Lewitus et al., 2016; DiCaro et al., 2019; Wang B. et al., 2019; Montesinos et al., 2020) (Tables 1, 2 and Supplementary Table 1) and 10 used a binge pattern protocol (Robson et al., 2013; Urrutia et al., 2014; Coelho-Santos et al., 2015; Frau et al., 2016; Ghanbari et al., 2019; Wang B. et al., 2019; Gou et al., 2020; Canedo et al., 2021; Hadizadeh-Bazaz et al., 2021; Kobeissy et al., 2022) (Tables 1, 2 and Supplementary Table 1). All these studies were conducted in rodent models.

The studies that used single exposure, evaluated only the central levels of cytokines. Upon methamphetamine (Meth) administration, several authors reported an increase in at least one of the classic pro-inflammatory cytokines TNF-α, IL-6, and IL-1β, in different brain regions [Nucleus accumbens (Nacc), the prefrontal cortex (PFC), the ventral tegmental area (VTA), the striatum and the hypothalamus] (Kelly et al., 2012; Seminerio et al., 2012; Nader et al., 2014; Frank et al., 2016; DiCaro et al., 2019) (Table 1). However, other studies reported that these cytokines were not altered in the hippocampus, PFC and Nacc, after a single administration of Meth or amphetamine (Amph) (DiCaro et al., 2019) (Table 1), and a few other studies reported asymmetric results across brain regions (Kelly et al., 2012; Gubert et al., 2016; Wang B. et al., 2019) (Table 1 and Supplementary Table 1). Only two studies addressed central cytokines after a single dose of cocaine, showing unaltered TNF-α levels in the ventral striatum and unaltered IL-1β in the hippocampus (Lewitus et al., 2016; Montesinos et al., 2020) (Table 3). Collectively, these studies evidence that drug-induced cytokine changes are region-dependent, which was confirmed by studies that evaluated the same cytokine in different regions (Kelly et al., 2012; Frank et al., 2016; Gubert et al., 2016; Wang B. et al., 2019) (see Table 1 and Supplementary Table 1). Of note, a number of other variables may also justify the differences observed between studies, such as differences in drug dosing, routes of administration, time-points of evaluation and the methodology used for cytokine quantification. These methodological differences are visible in Tables 1, 3 and Supplementary Table 1. Within these factors, dosing seems to strongly contribute to data heterogeneity, as studies that used low doses mostly showed no differences in cytokine levels (DiCaro et al., 2019; Wang B. et al., 2019) (Table 1), while studies that used higher doses showed increased TNF-α, IL-6 and IL-1β (Kelly et al., 2012; Seminerio et al., 2012; Nader et al., 2014; Frank et al., 2016) (Table 1).

Focusing on studies using binge patterns of administration, we found nine studies evaluating cytokines at the central level and just one evaluating circulating cytokine levels. In rodents, binge Meth administration resulted in cytokine changes similar to those reported for single dosing: (i) one study observed increased expression of TNF-α and IL-1β in both the striatum and the hippocampus (Canedo et al., 2021), (ii) two studies reported increased TNF-α (Coelho-Santos et al., 2015) or IL-6 in the striatum (Robson et al., 2013); and (iii) another study showed increased IL-6 and TNF-α in the mPFC, caudate putamen (CPu) and hippocampus (Gou et al., 2020) (Table 1). Additionally, two of the studies reviewed also reported persistently increased TNF-α levels past 7 and 12 days of Meth binge administration (Ghanbari et al., 2019; Hadizadeh-Bazaz et al., 2021) (Table 1). However, other authors reported no differences in the expression of TNF-α and IL-6 in regions like the hippocampus, PFC, CPu, and the Nacc (Wang B. et al., 2019; Canedo et al., 2021), as well as no differences in IL-1β in the striatum (Canedo et al., 2021) (Table 1).

Also, regarding binge administration but of MDMA, one work showed increased TNF-α and IL-1β levels in the CPu (Frau et al., 2016) (Supplementary Table 1).

As verified in the studies using single dosing, the heterogeneity of results observed for TNF-α, IL-6, and IL-1β under binge schedules were impacted by the same variables. Also in this case, studies using low doses mostly showed no impact in TNF-α, IL-6, and IL-1β levels (Wang B. et al., 2019; Canedo et al., 2021), while those using higher doses reported increased levels for these cytokines (Coelho-Santos et al., 2015; Ghanbari et al., 2019; Gou et al., 2020; Hadizadeh-Bazaz et al., 2021) (Table 1).

Additionally, two cytokines were evaluated in single studies (after Meth exposure): one study reported an increase in the expression of IL-15 in the striatum after binge administration (Urrutia et al., 2014) (Table 1) and one study reported no changes in IL-1α after single administration (DiCaro et al., 2019) (Table 1).

A single study evaluated circulating cytokines after binge Meth administration, reporting augmented expression of IL-1β, IL-6, and IL-10 (Kobeissy et al., 2022) (Table 2). Of note, within the pool of studies reviewed in this section, none evaluated central and peripheral levels of cytokines simultaneously.

Taken together, these results revealed that (i) nine (out of thirteen) studies evaluating TNF-α, showed increased levels; (ii) seven (out of nine) studies evaluating IL-1β reported increased levels; and (iii) five (out of eight) reported increase IL-6 in at least one brain region. Based on this, one can concluded that IL-6, TNF-α, and IL-1β were the most investigated cytokines in acute/binge exposure to psychoactive substances and that drug-induced variations in cytokines are region- and dose-dependent. As we found only a single study reporting on circulating cytokine levels, one can only concluded that more studies at the peripheral level are clearly necessary to understand how circulating cytokines vary with exposure to psychostimulants and how they relate to central cytokine levels.

### 3.3. Cytokine profile under short- and long-term administration of psychostimulants

In this section, we analyzed studies reporting on the effects of repeated administration (over a variable number of days) of psychoactive substances in cytokine levels, either at the central or peripherally. Importantly, repeated administration of psychostimulant may result in substance use disorder and unexpected system adaptations (Nestler and Luscher, 2019).

A total of 28 studies evaluating the short- and long-term effects of psychostimulant administration were considered. Eighteen reported on changes of cytokines in the brain, eight in the blood and two studies evaluated cytokines at both central and peripheral levels (Tables 1–5 and Supplementary Tables 1–3).

Goncalves et al. (2017) showed an increase in the expression of TNF-α in the striatum and hippocampus after seven days of self-administration, but not of IL-1β (Table 1). Other authors reported increased IL-1β in the hippocampus after 4 days of an escalating regimen of Meth (Liskiewicz et al., 2019) (Table 1). All four studies that used protocols of short- or long-term administration of other amphetamines, reported an increase in several cytokines such as TNF-α, IL-1β, IL-6, IFN-γ, IL-4, and IL-10, in the striatum or the frontal cortex (El-Sayed El-Sisi et al., 2016; Shin et al., 2016; Valvassori et al., 2018) (Supplementary Table 1). Gubert et al. (2016) reported that IL-1β and TNF-α levels were unaltered in the PFC and hippocampus after repeated amphetamine administration, while in the striatum TNF-α was also unaltered, but IL-1β was increased (Supplementary Table 1).

Studies that evaluated changes in central cytokines as a result of repeated cocaine exposure consistently showed increased IL-1β, TNF-α, and IL-6 (Lewitus et al., 2016; Mai et al., 2018; Montesinos et al., 2020; Chivero et al., 2021) and reduced IFN-γ (Mai et al., 2018) (Table 3). In these studies, performed in rodent models, the brain regions examined were the striatum, the cortex and the hippocampus (Lewitus et al., 2016; Mai et al., 2018; Montesinos et al., 2020; Chivero et al., 2021) (Table 3). Of note, Chivero et al. (2021) also showed higher IL-1β levels in human postmortem frontal cortices (Table 3).

Consubstantiating the results already reported for cocaine and amphetamines, several studies using rodent models of long-term administration of methylphenidate, also reported increased IL-1β and TNF-α in the hippocampus (Motaghinejad and Motevalian, 2016, 2022; Motaghinejad et al., 2016a,b,c, 2017; Schmitz et al., 2017; Ebrahimzadeh et al., 2019). Schmitz et al. (2017), further reported an increase in IL-6 levels in the hippocampus after methylphenidate administration (Table 5).

At the peripheral level, in rats, one study showed that TNF-α, IL-6, and IFN-γ levels were not affected by long-term Meth self-administration (Mata et al., 2015) (Table 2). Similarly, and also in rats, long-term administration of lisdexamfetamine, did not affect TNF-α, IL-1β, and IL-10 serum levels (Bristot et al., 2019) (Supplementary Table 2). However, in mice, repeated Meth administration resulted in increased circulating levels of TNF-α, IL-1β, and IL-18 (Wang et al., 2022) (Table 2).

Of note, Kuo et al. (2018) analyzed several cytokines in patients that met the criteria for Meth dependence and reported an increase of IL-1β, IL-2, IL-4, IL-6, and IL-10, 3 days after the last dosing (Table 2). This same study reported that IFN-γ, TNF-α, and IL-8 were not altered and that IL-5 was decreased (Kuo et al., 2018) (Table 2). Opposing to these results, other authors reported that IL-6 was not altered in the plasma and PBMCs of Meth-dependent patients with a recent dosing (72 h) (Jiang et al., 2016), while a another study presented increased TNF-α levels in the serum of Meth-dependent patients that were about to start a detoxification treatment (Shen et al., 2020) (Table 2).

Also at the peripheral level, studies that investigated variations of circulating IL-6 and IL-10 in patients with cocaine dependency reported either an increase in both cytokines relative to healthy participants (Pianca et al., 2017) or no changes in these two cytokines and TNF-α at the peripheral level (Ribeiro et al., 2021) (Table 4).

As already referred, only two studies addressed how psychostimulants affect cytokine levels at both peripheral and central levels. Both studies used a long-term administration of D-amphetamine in rats, and showed similar results for cortical, striatal (but not hippocampal) and circulating cytokines, i.e., increased levels of IL-4, IL-10, IL-6, and TNF-α (Valvassori et al., 2015, 2019). IL-1β was unchanged either at central or peripheral level (Valvassori et al., 2015, 2019) (Supplementary Table 3).

Taken together, these studies reveal that when evaluating short- and long-term effects of repeated exposure to psychostimulants, the target cytokines were again IL-6, TNF-α, and IL-1β. At the central level: (i) 13 out of 14 studies showed an increase in TNF-α levels in at least one brain region; (ii) four out of five studies reported increased IL-6, and (iii) all the twelve studies addressing IL-1β reported an increase. At the peripheral level: (i) two out of five studies presented an increase in TNF-α and IL-6, and (ii) two out of two studies reported an increase in IL-1β and IL-10. Interestingly, none of the two studies that evaluated the IFN-γ levels reported changes in this cytokine.

In summary, despite some variability, most of the cytokines evaluated seem to be increased in the brain after short and long-term exposure to psychostimulants, with more robust results for TNF-α, IL-6, and IL-1β. At the peripheral level there is a clear need for further investigation. Importantly, the few studies that analyzed cytokines both at the peripheral and central levels, reported similar results for the cytokines evaluated in both compartments.

### 3.4. Central and peripheral cytokine profiles at the withdrawal from psychostimulants

The withdrawal period encompasses a disruption in drug intake, in which one develops distressing feelings and strong physiologic reactions (Kuhar, 2012). A correct management of this period is critical to prevent drug relapse (Kuhar, 2012). In this context, understanding how cytokine levels impact on anxiety and impulsivity, critical factors for relapse, may contribute to more successful therapeutic approaches.

Within the pool of studies obtained for review that addressed the cytokine profile at the withdrawal from psychostimulants, we found 10 studies reporting on central cytokine levels, nine studies analyzing circulating levels, and one evaluating them both at central and peripheral levels (Tables 1–4 and Supplementary Tables 1, 3).

When considering studies that evaluated cytokines, at the central level, during withdrawal from Meth, five publications reported increased TNF-α in the hippocampus, striatum, substantia nigra and PFC (Jiang et al., 2014; Shin et al., 2016; Beirami et al., 2017; Goncalves et al., 2017; Namyen et al., 2020), while one publication showed decreased TNF-α in the frontal cortex and unaltered TNF-α in the amygdala and striatum (Stolyarova et al., 2015) (Table 1 and Supplementary Table 1). Other authors, also found unaltered TNF-α in the hypothalamus (Loftis et al., 2013) (Table 1). Of note, differently from other publications that resorted to shorter administration periods, i.e., 5–15 days (Jiang et al., 2014; Beirami et al., 2017; Goncalves et al., 2017; Namyen et al., 2020), Stolyarova et al. (2015) administered Meth for 4 weeks and evaluated cytokine levels 17 days after the last Meth administration, which may justify the divergences in reported data (Table 1).

Focusing on IL-1β, we identified two studies showing an increased expression in the substancia nigra, hippocampus and PFC (Jiang et al., 2014; Namyen et al., 2020), and two studies showing decreased IL-1β in the striatum and frontal cortex (Stolyarova et al., 2015; Goncalves et al., 2017) (Table 1). Additionally, three studies reported that IL-1β was unaltered in the hippocampus, hypothalamus, amygdala and striatum at the withdrawal from Meth (Loftis et al., 2013; Stolyarova et al., 2015; Goncalves et al., 2017) (Table 1). Again, these studies used different Meth doses (ranging from 0.3 to 20 mg/kg) and different administration periods, which invalidates direct comparisons.

Within the studies that evaluated the pro-inflammatory cytokine IL-6, one work showed increased levels in the hippocampus and PFC during the Meth withdrawal period (Beirami et al., 2017) (Table 1) and another showed its increase in striatum during withdrawal from para-methoxy-methamphetamine (Shin et al., 2016) (Supplementary Table 1). Other two studies reported that at Meth withdrawal IL-6 was unaltered in the hypothalamus, amygdala and striatum (Stolyarova et al., 2015; Namyen et al., 2020) (Table 1). Again, Stolyarova et al. (2015) reported the opposite effect in the PFC, showing a decrease in IL-6 expression during the withdrawal period (Table 1).

In addition, one of the publications analyzed, revealed unaltered levels of IFN-γ in the hypothalamus (Loftis et al., 2013) (Table 1), while Shin et al. (2016) (Supplementary Table 1) reported an increase in this cytokine in the striatum. Upon that, two studies reported unaltered IL-10 in the hypothalamus, amygdala and striatum (Loftis et al., 2013) and decreased IL-10 in the PFC after Meth withdrawal (Stolyarova et al., 2015) (Table 1). Lastly, IL-2 levels were seen increased in the hypothalamus (Loftis et al., 2013) (Table 1).

When addressing studies that evaluated cytokines during withdrawal from cocaine: (i) three studies reported unaltered TNF-α levels in the ventral striatum, hippocampus and mPFC (Lewitus et al., 2016; Mai et al., 2018; Zhu et al., 2018) (Table 3); (ii) one study reported that IL-6 was increased in the hippocampus (Mai et al., 2018); (iii) another that it was unaltered in the mPFC (Zhu et al., 2018); and this last study also showed that IL-1β levels were unaltered in the mPFC (Zhu et al., 2018) (Table 3). The levels of IFN-γ were described as decreased at earlier cocaine withdrawal (3 days after last administration) and as unaltered in longer periods of withdrawal (7 and 14 days after last administration) in the hippocampus (Mai et al., 2018) (Table 3).

At the peripheral level, opposing results were reported. In Meth abstinent patients, one study showed that IL-1β levels were unaltered in the plasma (Kohno et al., 2018), while another showed that IL-1β was increased (Kuo et al., 2018) (Table 2). The same was observed for TNF-α, one study reported unaltered levels (Kuo et al., 2018) and two studies reported an increase (Luo et al., 2022; Re et al., 2022) (Table 2). Opposing results were also described for IL-10, with Kohno et al. (2018) claiming that IL-10 was unaltered in Meth abstinent patients, and Kuo et al. (2018) reporting that IL-10 was decreased in the plasma of Meth abstinent patients (Table 2). Interestingly, Re et al. (2022), showed that IL-10 levels were unaltered in the plasma of patients in early periods of withdrawal (7–15 days and 3 months), but increased after 1 year of Meth withdrawal (Table 2).

For IL-6 and IL-2 reported data were consistent, showing an increase in these cytokines in three studies evaluating Meth abstinent patients (Kohno et al., 2018; Kuo et al., 2018; Re et al., 2022) (Table 2). Furthermore, for IL-2, similar results were observed in rats (Li et al., 2021) (Table 2).

Other cytokines were also evaluated in Meth abstinent patients, showing an increase in IL-4, IL-1α, IL-18, IL-12, IL-7, and IL-5, a decrease in IL-8 and IL-9, and unaltered levels of IFN-γ and IL-5 as measured in the serum (Kuo et al., 2018; Luo et al., 2022; Re et al., 2022) (Table 2). The strong level of variation observed among different studies may be attributable to differences in the target populations, since most were recruited from rehabilitation centers in China and Taiwan.

Regarding the studies performed in cocaine abstinent patients, one study evaluated the serum cytokine levels in adolescents, at different time-points of the withdrawal period, and observed that initially the levels of TNF-α and IL-6 were decreased, but after a longer period of abstinence, the levels of both cytokines were reestablished (Levandowski et al., 2016b) (Table 4). Another study, also in adolescents, showed unaltered IL-6 after a similar period of cocaine withdrawal in the serum (Pianca et al., 2017), and two studies reported an increase in IL-6 at a similar time-point, but in women meeting the criteria for cocaine (crack) dependence (Levandowski et al., 2016a; Zaparte et al., 2019) (Table 4). Yet in cocaine abstinent patients, different studies addressing circulating levels reported: (i) an increase in TNF-α (Zaparte et al., 2019); (ii) increased IL-10 in adults (Levandowski et al., 2016b; Zaparte et al., 2019); (iii) unaltered IL-10 in adolescents (Pianca et al., 2017); (iv) increased IFN-γ, IL-2 and IL-17 in PBMCs (Zaparte et al., 2019); (v) decrease IFN-γ, IL-2 and IL-17 in the serum (Levandowski et al., 2016b); and (vi) augmented IL-4 in two studies (Levandowski et al., 2016b; Zaparte et al., 2019) (Table 4).

A single study addressed cytokines both at central and peripheral levels at withdrawal, using a binge protocol of amphetamine (3 × 1 mg/kg, 3 h apart) for six consecutive days, and reporting increased TNF in the striatum and in serum, and unaltered TNF-α in the hippocampus (You et al., 2020) (Supplementary Table 3).

**Figure 3:**
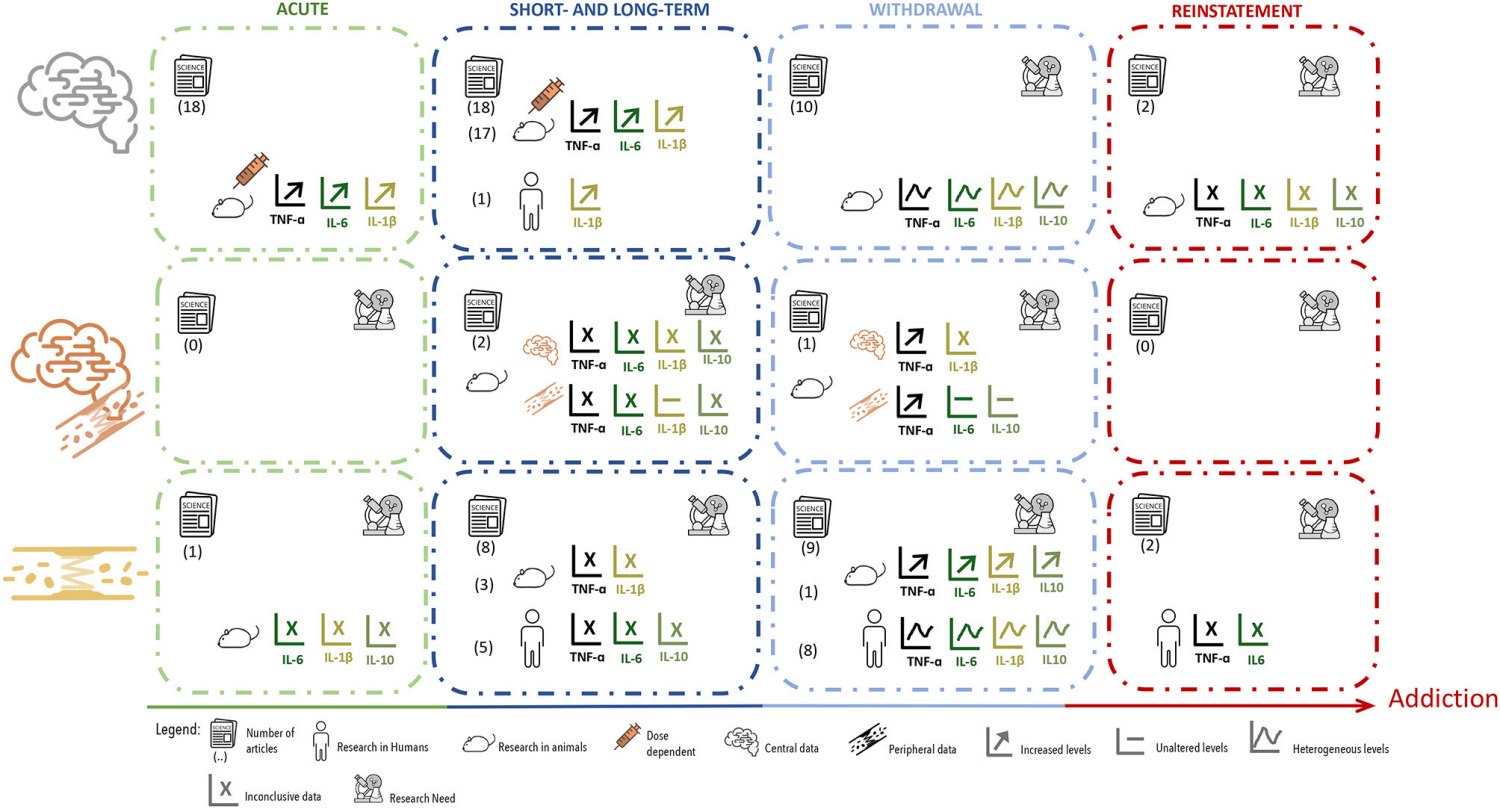
Summary of data distribution according to cytokines measurement, showing the number, type and major results of manuscripts that evaluated cytokines in brain tissue, brain tissue plus blood or serum. Manuscripts were further divided according to the type of exposure and timepoint of evaluation.

Taken together, at central level: (i) five out of 10 studies showed an increase in TNF-α and only one reported decreased levels; (ii) four out of seven showed increased IL-6 and IL-1β; and (iii) one out of two presented a decrease in IL-10 levels. At the peripheral level, (i) three out of six studies reported an increase in TNF-α: (ii) six out of eight showed an increase in IL-6; (iii) one out of three reported increased IL-1β; (iv) two out of seven presented increased IL-10; (v) two out of four showed an increase in IFN-γ; (vi) three out of four presented increased IL-2; and (vii) one out of two reported increased IL-17 (see also Figure 3). Up on that, four studies evaluated peripheral levels of IL-4, and all reported increased levels. For all the cytokines evaluated at peripheral level, with an exception of IL-4, only one study reported decreased levels.

As described above, the pool of studies that reported data on how withdrawal from psychostimulants affect the cytokine profile, described very heterogeneous results. These may be influenced by several parameters that differ between the studies such as dosing, age of use, or other unreported pathologies; however, the period of withdrawal in which cytokines were evaluated seems to be a powerful modulator of the outcome. This is confirmed in studies that evaluated cytokine profiles in different withdrawal periods and timepoints (Jiang et al., 2014; Levandowski et al., 2016b; Mai et al., 2018; Re et al., 2022).

### 3.5. Central and peripheral cytokines profiles after psychostimulant reinstatement

The high rates of relapse are a major obstacle in the treatment of drug addiction (Kuhar, 2012). Therefore, it is important to address also how relapse itself impacts on cytokines. This can be nicely investigated using animal models of reinstatement (Kuhar, 2012), which are considered a measure of relapse in drug intake.

Among the four studies that evaluated cytokines after psychostimulants reinstatement, two studies measured cytokines in the brain (Brown et al., 2018; Karimi-Haghighi et al., 2020) and two studies in the blood (Gupta et al., 2018; Li et al., 2021) (Tables 1–4). No studies evaluating simultaneously cytokines at central and peripheral level were found.

One of the reviewed studies evaluated the levels of TNF-α, IL-10, IL-1β, and IL-6 in the PFC and hippocampus of rodents, after reinstatement with two different doses of Meth (0.25 or 0.5 mg/Kg) (Karimi-Haghighi et al., 2020) (Table 1). The levels of TNF-α and IL-10 were increased in both brain regions after reinstatement following the highest Meth dose. The levels of IL-1β were increased with both reinstatement doses, but only in the PFC. No changes were reported for IL-6. At the central level, another study, also in rodents, showed increased IL-1β in the VTA after a cocaine challenge, but no differences in TNF-α levels (Brown et al., 2018) (Table 3).

Lastly, Li et al. (2020) showed that in patients suffering from Meth use disorder, a Meth challenging-dose increased IL-6 levels within 360 min of Meth infusion (Table 2). The same study showed that TNF-α levels were not affected at that time point (Li et al., 2020). Gupta et al. (2018), on the other hand, showed in adult cocaine users, that a cocaine challenging does not seem to affect IL-6 levels in the serum at 6 h, 24 h, and 6 days past cocaine (Table 4).

Based on the reduced number of studies addressing cytokines at reinstatement and the variability on reported data, the only possible conclusion is that a strong research effort is yet necessary in the field.

## 4. Discussion

There is robust evidence confirming that psychostimulants strongly impact on cytokine production and release both at central and peripheral level. However, a number of important matters remain unclear, of which we highlight that: (i) there is a clear lack of information regarding the temporal course of cytokines dysregulation along the transition from episodic to problematic use and progression to addiction; (ii) the crosstalk between central and peripheral immune players, under exposure to psychostimulants, and whether this crosstalk could be a valuable therapeutic target, remains elusive. The present scoping review aimed at providing a comprehensive compilation of the cytokine profile at central and peripheral level in the different stages of the addictive process, envisioning the understanding of the crosstalk between these two compartments of the immune system during the disease progression and, eventually the use of cytokines as biomarkers. From the present analysis, it is possible to conclude that the available data are mostly focused on the classical pro-inflammatory cytokines TNF-α, IL-6, and IL-1β. This was not an unexpected result since, in general, psychoactive substances have been described to activate pro-inflammatory immune responses and inflammation has been associated with drug-seeking, craving and withdrawal (Crews et al., 2011; Cui et al., 2014; Harricharan et al., 2017).

Focusing on the studies that evaluated TNF-α in the brain, all of them conducted in animal models, and looking into the results longitudinally, throughout the different phases of the addictive process, this cytokine was seen increased at central level, mainly after acute exposures and during the short- and long-term administration of psychostimulants (Figure 3). Contrarily, during the withdrawal period, the results were heterogeneous, with studies showing changes in TNF-α levels in opposite directions (Figure 3). Additionally, the results for TNF-α levels at the periphery were less robust, which can simply be a consequence of fewer studies having evaluated this cytokine in the blood, or because they were evaluated in different groups (such adult women vs. adolescents) (Figure 3). Only further research will clarify this issue. The differences observed for TNF-α levels after either acute or after short- and long-term exposure to psychostimulants, may be justified by several variables, such as differences in drug dosing, route of administration or time-point of evaluation. Furthermore, at the central level, these differences were highly dependent on the brain region evaluated. At the withdrawal period, however, one can speculate that the heterogeneous results reported were mostly due to differences in timepoints of analysis (WHO, 2009).

Over the past few years, cytokines have been described as modulators of behavior (reviewd in Salvador et al., 2021). In problematic drug use they seem to vary according to the stage of the disease and consequently differently impact on behavior throughout disease progression. In particular, TNF-α has been described as an important modulator of different behaviors, such as sickness behavior, depressive-like behavior and cognitive dysfunction (Kaster et al., 2012; Hennessy et al., 2017; Fourrier et al., 2019; Salvador et al., 2021). Moreover, several studies suggest that TNF-α produced by peripheral immune cells, may signal CNS cells and impact on different behaviors (Kaster et al., 2012; Hennessy et al., 2017; Salvador et al., 2021). Recently, it was demonstrated that circulating TNF-α levels were positively associated with depression scores in people with drug use disorder (Martinez et al., 2018), while dampened TNF-α and TNFR1 levels were associated with stress response in abstinent alcohol-dependent individuals (Fox et al., 2017). Additionally, the severity of withdrawal symptoms was positively associated with TNF-α levels (Fox et al., 2017). As such, it is possible that under psychostimulants, increased TNF-α, acutely and after longer periods of exposure, may be positively related with depressive mood. Likewise, TNF-α variable levels during abstinent periods may also be associated with variable withdrawal symptoms and increased likelihood of relapse. However, the scarcity of results regarding TNF-α levels at drug reinstatement averts a deeper understanding of its possible role in relapse.

The pro-inflammatory cytokine IL-6 was investigated in fewer studies than TNF-α, but looking into the results longitudinally, allows perceiving that these two cytokines display similar variations across the different phases of exposure to psychostimulants (Figure 3). Centrally, the levels of IL-6 were reported, in animal models, as mainly increased under acute exposure or short- to long-term administration of psychostimulants; while during the withdrawal period the results were more heterogeneous (Figure 3). When a addressing the variation of circulating IL-6, again, in different human populations results were variable. Furthermore, there is, to some extent, higher heterogeneity in the levels of IL-6 reported within each period, which may be explained by the factors already mentioned for TNF-α.

In the CNS, IL-6 signaling modulates a variety of stress-related and sickness-like behaviors (Barney et al., 2019; Salvador et al., 2021). A growing body of evidence suggests that IL-6 has a crucial role in the pathogenesis of depression (Barney et al., 2019) and recent studies, both pre-clinical and clinical, demonstrated a functional role for IL-6 in the development of major depressive disorder (MDD) (Roohi et al., 2021). In MDD patients, IL-6 is the most consistently increased cytokine in the blood (Dowlati et al., 2010; Miranda et al., 2018; Barney et al., 2019), which was also reported as increased at the central level (Kern et al., 2014). This cytokine has been suggested as a promising potential target to treat depression (Roohi et al., 2021). In pathological alcohol use, IL-6 was positively associated with depression and psychological distress scores (Martinez et al., 2018). Moreover, elevated concentrations of IL-6 at peripheral level have been associated with cognitive decline (Mooijaart et al., 2013; Tegeler et al., 2016) and with Meth-induced mesocorticolimbic deficits (Kohno et al., 2018). Nevertheless, further studies are still necessary to understand the role of IL-6 in depressive behavior and impaired cognition, which are also hallmarks of the problematic use of psychostimulants.

Focusing into the results obtained for IL-1β, we observe that, centrally (and therefore mostly in animal models), the levels of this cytokine were reported as increased in at least one brain region, in most studies using acute administration of psychostimulants and in all studies performed after short- or long-term administration. At peripheral level, IL-1β was augmented in the few studies performed. When evaluated at the withdrawal, most studies also showed higher levels of IL-1β in at least one brain region, but circulating levels were very inconsistent, calling for further research. This inconsistency is again driven by a reduced number of studies, conducted in very different groups of patients.

IL-1β has been described as an important modulator of sickness behavior and cognition and seems to be critical for maintaining homeostatic sleep behavior (Salvador et al., 2021). Regarding cognition, in a mice model for Alzheimer disease, a chronic systemic administration of anti-IL-1R, in mice, resolved brain inflammation and reversed cognitive deficits (Kitazawa et al., 2011). Another study suggested that increased serum levels of IL-1β were a stage marker for brain neurodegenerative progression (Forlenza et al., 2009). In accordance, one of the articles reviewed reported that cognitive decline after Meth exposure could be associated with IL-1 levels in the hippocampus (Liskiewicz et al., 2019). Additionally, blockade of IL-1β signaling can lead to sleep disruption (Opp and Krueger, 1994; Takahashi et al., 1997), which will also affect memory consolidation. As such, further studies focusing on IL-1β levels at the periphery and its contribution for behavioral disruption in drug context will be highly relevant.

Considering the results for the classical pro-inflammatory cytokines, TNF-α, IL-6, and IL-1β, one could conclude that the majority of the studies showed an increase in these cytokines at central level. However, peripheral results are scarcer and much less consistent. Therefore, further studies will be necessary to understand the crosstalk between the two immune compartments in the context of disease progression. Moreover, there is a demand for longitudinal studies with measurements, starting in the acute phase of psychostimulant exposure, covering different moments of long-term exposure, and addressing several time-points of withdrawal and reinstatement. Such data sets will finally clarify how TNF-α, IL-6, and IL-1β vary across the progression from occasional use to addiction.

Although, most studies included in this scoping review were focused on pro-inflammatory cytokines, a considerable number of studies also evaluated the anti-inflammatory cytokine IL-10. In the reviewed literature, IL-10 levels were mainly evaluated at the periphery and the results were heterogeneous. Contrary to what one could expect, acute, short- and long-term administration of psychostimulants resulted in increased levels of IL-10. The same was seen after reinstatement. Augmented IL-10 may be justified as a response to the elevation of pro-inflammatory cytokines (Pianca et al., 2017), in particular to IFN- γ as recently described (Cardoso et al., 2021). On the other hand, the heterogeneous results observed during the withdrawal period may be a consequence of sampling cytokines at variable time points, as already discussed.

Variations in IL-10 expression are associated with alterations in depressive-like behavior, but do not seem to influence cognitive performance (Mesquita et al., 2008). Importantly, in users diagnosed with alcohol disorder, IL-10 was negatively associated with anxiety scores (Martinez et al., 2018). Moreover, overexpressing IL-10, specifically in the NAcc, reduced self-administration of remifentanil in rats (Lacagnina et al., 2017), and injections of recombinant IL-10 into the basolateral amygdala during a Drinking in the Dark (DID) paradigm attenuated binge-like ethanol consumption in mice (Marshall et al., 2017). These observations suggest that IL-10 could be an important target for the treatment of addiction.

Despite some of the reviewed studies having evaluated IFN-γ and IL-4, available data is still very limited, which difficult a better understanding of the role of these cytokines in the addictive process. However, further studies focusing on the role of both cytokines will be of great importance. IFN-γ was described as an important modulator of social behavior (Filiano et al., 2016), which is impaired during the psychostimulant abstinent period (Favrod-Coune and Broers, 2010). Additionally, IL-4 was described as a critical player in learning and memory (Gadani et al., 2012) and also participating in the regulation of depressive-like behavior (Wachholz et al., 2017).

### 4.1. Limitations

As already discussed this scoping review is limited by a reduced number of studies addressing cytokines simultaneously at central and peripheral levels and by methodology differences. However, it is also important to highlight that drug users are highly heterogenous populations, not always sufficiently characterized, and most likely suffering from stress and mood disorders that can also affect the cytokine profile and impact on the data reported (Montagud-Romero et al., 2022). Sex differences can also strongly affect cytokine profiles, which was not explored. Upon that, several studies have also identified chemokines as possible immunoregulators and this is not addressed in the present study.

### 4.2. Conclusion

In summary, a wide use of arrays for several cytokines should be strongly considered to better determine which cytokines, upon the classical ones, may be involved in the progression from episodic use to the development of addiction. Additionally, a concerted effort should be accomplished to better understand the link between peripheral and central immune players in longitudinal expression profiles of cytokines and its dynamics under exposure to different psychoactive substances.

## Data availability statement

The original contributions presented in the study are included in the article/Supplementary material, further inquiries can be directed to the corresponding author.

## Author contributions

Conceptualization: JB, AM, and TS. Formal analysis: JB and CM. Funding acquisition, project administration, and supervision: TS. Investigation: JB, CM, and EA. Methodology and writing—review and editing: JB and TS. Writing—original draft: JB, EA, AM, and TS. All authors have read and agreed to the published version of the manuscript.
