# Supplementary material for: The impact of psychostimulants on central and peripheral neuro-immune regulation: a scoping review of cytokine profiles and their implications for addiction
Source: Front Cell Neurosci. 2023 May 26;17:1109611. doi: 10.3389/fncel.2023.1109611 (PMC10251407; doi:10.3389/fncel.2023.1109611)
Supplement: Supplementary file 1 [file Table_1.docx]

Supplementary Material

**Supplementary material for Bravo et al. The impact of psychostimulants on central and peripheral neuro-immune regulation: A scoping review cytokine profiles and their implications for addiction**

**Includes:**

**Supplementary Table 1, Supplementary Table 2 and Supplementary Table 3**

**Supplementary Table 1: Studies addressing the impact of amphetamines (other than Meth) in central cytokines**

|  | References | Subjects | Substance | Dose regimen | Evaluation | Tissue | Results | Methodology |
| --- | --- | --- | --- | --- | --- | --- | --- | --- |
| Acute | (39) Frau et al, 2016 | Adult and adolescent male C57BL/6J mice | MDMA | 4x20 mg/Kg, 2 h apart, i.p. | 48 h after administration | CPu | Adults  IL-1β and TNF  (no differences in the adolescent mice group) | Immuno-  histochemistry |
|  | (32) Gubert et al., 2016 | Adult male C57BL/6J mice | Amphetamine | A single injection of2 mg/Kg, i.p. | After last injection | PFC  Striatum  Hippocampus | PFC and hippocampus  = IL-1β and TNF  Striatum  IL-1β  = TNF | Flow Cytometric bead array |
| Short- and long-term | (32) Gubert et al., 2016 | Adult male C57BL/6J mice | Amphetamine | 2 mg/Kg once a day for 7 days, i.p. | After last injection | PFC  Striatum  Hippocampus | PFC and hippocampus  = IL-1β and TNF  Striatum  IL-1β  = TNF | Flow Cytometric bead array |
|  | (43) El-Sayed El-Sisi et al., 2016 | Adult male rats | Amphetamine | 2 mg/Kg, for 5 days, s.c. | 24 h after the last injection | Brain | TNF | ELISA |
|  | (45) Valvassori et al., 2018 | Male Wistar rats | Dextroamphetamine | 2mg/Kg once a day for 14 days, i.p. | At day 15 | Frontal cortex and striatum | Cortex and striatum  IL-4; IL-6, IL-10 and TNF | ELISA |
|  | (44) Shin et al., 2016 | C57BL/6J mice | Para-methoxy  -methamphetamine | 60mg/Kg, twice a day for 4 consecutive days. | 1 h; 2 h; 1 day and 7 days | Striatum | IL-6 (at 1h; 12h and 7 days)  IFN-γ and TNF | RT-PCR and western blot |

Abbreviations: CPu – Caudate putamen; ELISA - enzyme-linked immunosorbent assay; IFN – Interferon; IL – Interleukin; i.p. – intraperitoneal; PFC – Prefrontal cortex; RT-PCR – Real Time Polymerase Chain Reaction; s.c. – subcutaneous; TNF – Tumor necrosis factor. Results are expressed relative to control ( - increase; = - unaltered).

**Supplementary Table 2: Studies addressing the impact of amphetamines (other than Meth) in peripheral cytokines**

|  | References | Subjects | Substance | Dose regimen | Evaluation | Tissue | Results | Methodology |
| --- | --- | --- | --- | --- | --- | --- | --- | --- |
| Short- and long-term | (56) Bristot et al., 2019 | Adult male Wistar rats | Lisdexamfetamine | One daily administration of 10 mg/Kg, for 14 days, gavage | On the day 14 | Serum | = TNF, IL-1β and IL-10 | Milliplex Map Kit |

Abbreviations: IL – Interleukin; TNF – Tumor necrosis factor. Results are expressed relative to control (= - unaltered).

**Supplementary Table 3: Studies addressing the impact of amphetamines (other than Meth) in both peripheral and central cytokines**

|  | References | Subjects | Substance | Dose regimen | Evaluation | Tissue | Results | Methodology | |
| --- | --- | --- | --- | --- | --- | --- | --- | --- | --- |
| Short- and long-term | (62) Valvassori et al., 2019 | Male Wistar rats | Dextroamphetamine | 2mg/Kg once a day for 14 days, i.p. | At day 15 | Serum, cortex and striatum | Serum, cortex and striatum  = IL1-β  IL-4 and IL-10 | | ELISA |
|  | (63) Valvassori et al., 2015 | Male Wistar rats | Dextroamphetamine | 2mg/Kg once a day for 14 days, i.p. | At day 15 | Serum, CSF, frontal cortex, hippocampus and striatum | Serum, cortex and striatum  = IL1-β  IL-4, IL-6, IL-10 and TNF  Hippocampus and CSF  = IL1-β, IL-4, IL-6, IL-10 and TNF | | ELISA |
| Withdrawal | (77) You et al., 2020 | Male C57BL/6J mice | Amphetamine | Binge amphetamine 3x1 mg/kg (3h apart, 3xAMP) for 6 consecutive days, or 3 daily administrations, using an escalating dose regimen, consisting of 60 administrations ranging from 1 to 10 mg/Kg. | 21 days past 6 days of Meth or 21 days past 20 days of escalating Meth (both with stressful behavioral evaluation in between). | Striatum, hippocampus, lymph nodes and serum. | 3xAMP:  Striatum  TNF and IL-1β  Hippocampus  = TNF and IL-1β  Serum  TNF  = IL-10, IL-6, IL-4 and IFN-γ  Escalating:  Striatum  TNF  = IL-1β  Hippocampus  TNF  = IL-1β | | RT-PCR and Bio-Plex® |

Abbreviations: AMP – Amphetamine; CSF - Cerebrospinal fluid; IFN – Interferon; ELISA - enzyme-linked immunosorbent assay; IL – Interleukin; i.p. – intraperitoneal; RT-PCR – Real Time Polymerase Chain Reaction; TNF – Tumor necrosis factor. Results are expressed relative to control ( - increase; = - unaltered).
